# 3-(3-Cyano­benz­yl)-1-methyl-1*H*-imidazol-3-ium hexa­fluoro­phosphate

**DOI:** 10.1107/S1600536812001882

**Published:** 2012-01-21

**Authors:** Rosenani A. Haque, Zulikha H. Zetty, Abbas Washeel Salman, Hoong-Kun Fun, Chin Wei Ooi

**Affiliations:** a School of Chemical Sciences, Universiti Sains Malaysia, 11800 Penang, Malaysia; b X-ray Crystallography Unit, School of Physics, Universiti Sains Malaysia, 11800 USM, Penang, Malaysia

## Abstract

In the title compound, C_12_H_12_N_3_^+^·PF_6_^−^, the hexa­fluoro­phosphate anion is disordered over two orientations with refined site occupancies of 0.8071 (17) and 0.1929 (17). The dihedral angle between the imidazole and benzene rings in the cation is 71.26 (7)°. In the crystal, the cations and anions are linked by C—H⋯F and C—H⋯N hydrogen bonds into a three-dimensional network.

## Related literature

For details and applications of *N*-heterocyclic carbenes, see: Hermann *et al.* (1997 ▶); Wanzlick & Kleiner (1961 ▶); Hermann & Köcher (1997 ▶); Baker *et al.* (2007 ▶); Gade & Laponnaz (2007 ▶); Özdemir *et al.* (2005 ▶); Köcher & Hermann (1997 ▶); Cetinkaya *et al.* (1997 ▶). For a related structure, see: Haque *et al.* (2011 ▶). For bond-length data, see: Allen *et al.* (1987 ▶). For the stability of the temperature controller used in the data collection, see: Cosier & Glazer (1986 ▶).
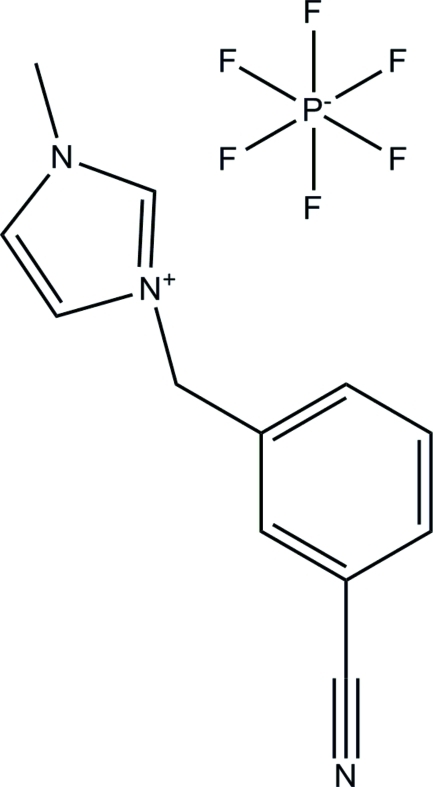


## Experimental

### 

#### Crystal data

C_12_H_12_N_3_^+^·F_6_P^−^
*M* *_r_* = 343.22
Triclinic, 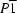

*a* = 5.9782 (1) Å
*b* = 8.7920 (1) Å
*c* = 14.1028 (2) Å
α = 77.975 (1)°
β = 83.279 (1)°
γ = 86.635 (1)°
*V* = 719.55 (2) Å^3^
*Z* = 2
Mo *K*α radiation
μ = 0.26 mm^−1^
*T* = 100 K
0.43 × 0.24 × 0.21 mm

#### Data collection

Bruker SMART APEXII CCD diffractometer
Absorption correction: multi-scan (*SADABS*; Bruker, 2009 ▶) *T*_min_ = 0.897, *T*_max_ = 0.949
14376 measured reflections
5233 independent reflections
4485 reflections with *I* > 2σ(*I*)
*R*_int_ = 0.019

#### Refinement

*R*[*F* ^2^ > 2σ(*F* ^2^)] = 0.047
*wR*(*F* ^2^) = 0.123
*S* = 1.04
5233 reflections
246 parameters
21 restraints
H-atom parameters constrained
Δρ_max_ = 0.91 e Å^−3^
Δρ_min_ = −0.64 e Å^−3^

### 

Data collection: *APEX2* (Bruker, 2009 ▶); cell refinement: *SAINT* (Bruker, 2009 ▶); data reduction: *SAINT*; program(s) used to solve structure: *SHELXTL* (Sheldrick, 2008 ▶); program(s) used to refine structure: *SHELXTL*; molecular graphics: *SHELXTL*; software used to prepare material for publication: *SHELXTL* and *PLATON* (Spek, 2009 ▶).

## Supplementary Material

Crystal structure: contains datablock(s) global, I. DOI: 10.1107/S1600536812001882/hb6605sup1.cif

Structure factors: contains datablock(s) I. DOI: 10.1107/S1600536812001882/hb6605Isup2.hkl

Supplementary material file. DOI: 10.1107/S1600536812001882/hb6605Isup3.cml

Additional supplementary materials:  crystallographic information; 3D view; checkCIF report

## Figures and Tables

**Table 1:** Hydrogen-bond geometry (Å, °)

*D*—H⋯*A*	*D*—H	H⋯*A*	*D*⋯*A*	*D*—H⋯*A*
C1—H1*A*⋯F1	0.95	2.46	3.240 (2)	139
C1—H1*A*⋯F5	0.95	2.27	3.1715 (18)	159
C2—H2*A*⋯F3^i^	0.95	2.46	3.250 (2)	140
C3—H3*A*⋯N3^ii^	0.95	2.49	3.3970 (19)	160
C4—H4*B*⋯F4^iii^	0.99	2.44	3.177 (2)	131
C6—H6*A*⋯F4	0.95	2.43	3.361 (2)	167
C10—H10*A*⋯N3^ii^	0.95	2.56	3.5019 (17)	170
C11—H11*B*⋯F6^iv^	0.98	2.53	3.400 (2)	148
C11—H11*C*⋯F1^v^	0.98	2.41	3.353 (2)	162

Symmetry codes: (i) 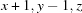; (ii) 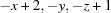; (iii) 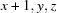; (iv) 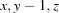; (v) 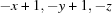.

**Fig. 1.**
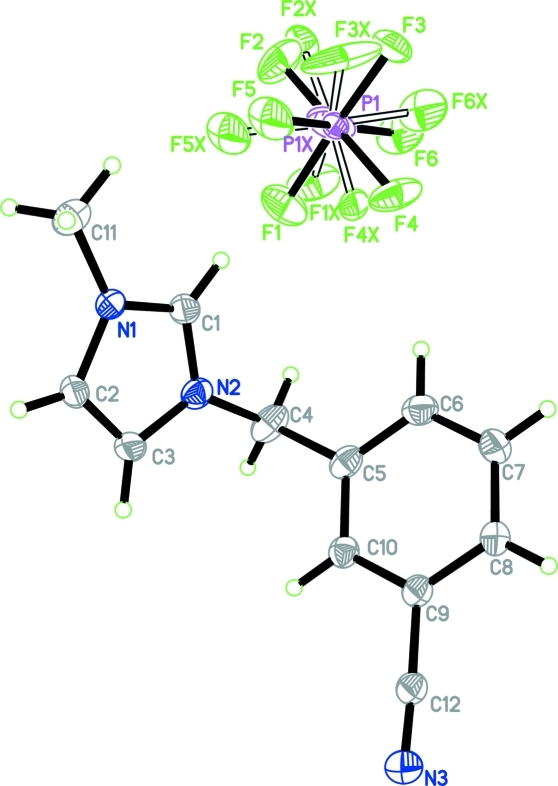
The molecular structure of the title compound, showing 50% probability displacement ellipsoids. The minor component is shown by the open bonds.

**Fig. 2.**
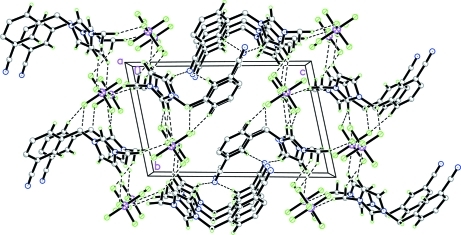
The crystal packing of the title compound, showing the three-dimensional network. Only the major component is shown. H atoms not involved in the intermolecular interactions (dashed lines) have been omitted for clarity.

## supplementary crystallographic information

### Comment

N-heterocyclic carbenes (NHC) are stable singlet carbenes that are most frequently prepared *via* deprotonation of azolium salts. Their role as ligands in coordination and modern organometallic synthesis have been widespread, since the investigation of NHC chemistry by Wanzlick in the early 1960s. NHCs have been proven as an alternative to tertiary phosphines in homogeneous catalysis for the past decades, as they have the ability to bond with metals in a variety of oxidation states through their strong σ-donating and negligible π-accepting characters. Furthermore, they are easy to handle and have been shown to be remarkably stable towards air and moisture. NHC complexes with every transition metals have received considerable attention and their diverse applications particularly in the area of catalysis such as olefin metathesis, transfer hydrogenation, hydroformylation, and furan synthesis have been investigated.

The asymmetric unit of the title compound (Fig. 1) consists a 3-(3-cyanobenzyl)-1-methylimidazolium cation and a hexafluorophosphate anion. The hexafluorophosphate anion is disordered over two orientations with refined site occupancies of 0.8071 (17) and 0.1929 (17). The imidazole ring (N1/N2/C1–C3) is essentially planar with a maximum deviation of 0.003 (1) Å at atom N2. The dihedral angle between the imidazole ring and the benzene ring (C5–C10) is 71.26 (7)°. The bond lengths (Allen *et al.*, 1987) and angles are within normal ranges and are comparable to the related structure (Haque *et al.*, 2011).

In the crystal structure (Fig. 2), the cations and anions are linked *via* C1—H1A···F1, C1—H1A···F5, C2—H2A···F3, C3—H3A···N3, C4—H4B···F4, C6—H6A···F4, C10—H10A···N3, C11—H11B···F6, and C11—H11C···F1 hydrogen bonds (Table 1) into a three-dimensional network.

### Experimental

To a solution of 1-methylimidazole (0.9 g, 11.0 mmol) in 25 ml of 1,4-dioxane, 3-(bromomethyl)benzonitrile (2.2 g, 11.0 mmol) was added. The mixture was refluxed at 90 °C overnight. The product was isolated by removing the solvent under reduced pressure, and then washed with fresh 1,4-dioxane (2 *x* 3 ml). The resulting bromide salt was converted quantitatively to its hexafluorophosphate counterpart by metathesis reaction using KPF_6_ (1.9 g, 10.3 mmol) in 25 ml of methanol. The white precipitate was collected and washed with distilled water (2 *x* 3 ml) and then left to dry at ambient temperature. Yield: 1.7 g, (48%); *M.p*: 104–106 °C. Colourless blocks were obtained by slow evaporation of the salt solution in acetonitrile at ambient temperature.

### Refinement

All the H atoms were positioned geometrically and refined using a riding model with *U*_iso_(H) = 1.2 or 1.5*U*_eq_(C) (C—H = 0.95, 0.98 or 0.99 Å). A rotating group model was applied to the methyl group. The hexafluorophosphate anion is disordered over two orientations with refined site occupancies of 0.8071 (17) and 0.1929 (17). In the final refinement, the outliners (5 - 4 9) and (5 - 2 10) were omitted. The same *U*_ij_ parameters were used for atom pairs F5/F5X, F6/F6X and C9/C12.

### Crystal data

**Table:**

C_12_H_12_N_3_^+^·F_6_P^−^	*Z* = 2
*M**_r_* = 343.22	*F*(000) = 348
Triclinic, *P*1	*D*_x_ = 1.584 Mg m^−^^3^
Hall symbol: -P 1	Mo *K*α radiation, λ = 0.71073 Å
*a* = 5.9782 (1) Å	Cell parameters from 6359 reflections
*b* = 8.7920 (1) Å	θ = 2.5–32.7°
*c* = 14.1028 (2) Å	µ = 0.26 mm^−^^1^
α = 77.975 (1)°	*T* = 100 K
β = 83.279 (1)°	Block, colourless
γ = 86.635 (1)°	0.43 × 0.24 × 0.21 mm
*V* = 719.55 (2) Å^3^	

### Data collection

**Table:**

Bruker SMART APEXII CCD diffractometer	5233 independent reflections
Radiation source: fine-focus sealed tube	4485 reflections with *I* > 2σ(*I*)
graphite	*R*_int_ = 0.019
φ and ω scans	θ_max_ = 32.7°, θ_min_ = 2.4°
Absorption correction: multi-scan (*SADABS*; Bruker, 2009)	*h* = −8→8
*T*_min_ = 0.897, *T*_max_ = 0.949	*k* = −13→13
14376 measured reflections	*l* = −21→21

### Refinement

**Table:**

Refinement on *F*^2^	Primary atom site location: structure-invariant direct methods
Least-squares matrix: full	Secondary atom site location: difference Fourier map
*R*[*F*^2^ > 2σ(*F*^2^)] = 0.047	Hydrogen site location: inferred from neighbouring sites
*wR*(*F*^2^) = 0.123	H-atom parameters constrained
*S* = 1.04	*w* = 1/[σ^2^(*F*_o_^2^) + (0.0569*P*)^2^ + 0.372*P*] where *P* = (*F*_o_^2^ + 2*F*_c_^2^)/3
5233 reflections	(Δ/σ)_max_ = 0.001
246 parameters	Δρ_max_ = 0.91 e Å^−^^3^
21 restraints	Δρ_min_ = −0.64 e Å^−^^3^

### Special details

**Table:**

Experimental. The crystal was placed in the cold stream of an Oxford Cryosystems Cobra open-flow nitrogen cryostat (Cosier & Glazer, 1986) operating at 100.0 (1) K.
Geometry. All e.s.d.'s (except the e.s.d. in the dihedral angle between two l.s. planes) are estimated using the full covariance matrix. The cell e.s.d.'s are taken into account individually in the estimation of e.s.d.'s in distances, angles and torsion angles; correlations between e.s.d.'s in cell parameters are only used when they are defined by crystal symmetry. An approximate (isotropic) treatment of cell e.s.d.'s is used for estimating e.s.d.'s involving l.s. planes.
Refinement. Refinement of *F*^2^ against ALL reflections. The weighted *R*-factor *wR* and goodness of fit *S* are based on *F*^2^, conventional *R*-factors *R* are based on *F*, with *F* set to zero for negative *F*^2^. The threshold expression of *F*^2^ > σ(*F*^2^) is used only for calculating *R*-factors(gt) *etc*. and is not relevant to the choice of reflections for refinement. *R*-factors based on *F*^2^ are statistically about twice as large as those based on *F*, and *R*- factors based on ALL data will be even larger.

### Fractional atomic coordinates and isotropic or equivalent isotropic displacement parameters (Å^2^)

**Table:**

	*x*	*y*	*z*	*U*_iso_*/*U*_eq_	Occ. (<1)
P1	0.0915 (2)	0.73349 (10)	0.16370 (8)	0.0173 (2)	0.8071 (17)
F1	0.3566 (2)	0.7033 (2)	0.15625 (12)	0.0487 (4)	0.8071 (17)
F2	0.1025 (4)	0.8343 (3)	0.05737 (16)	0.0487 (6)	0.8071 (17)
F3	−0.1762 (2)	0.75757 (17)	0.17710 (10)	0.0374 (3)	0.8071 (17)
F4	0.0797 (3)	0.6287 (2)	0.27361 (12)	0.0451 (4)	0.8071 (17)
F5	0.0591 (2)	0.57833 (15)	0.12519 (12)	0.0465 (4)	0.8071 (17)
F6	0.1151 (3)	0.88412 (15)	0.20813 (11)	0.0484 (4)	0.8071 (17)
P1X	0.0958 (11)	0.7363 (8)	0.1577 (5)	0.045 (2)	0.1929 (17)
F1X	0.3348 (10)	0.8099 (9)	0.1558 (4)	0.0486 (16)	0.1929 (17)
F2X	0.0465 (16)	0.8713 (10)	0.0645 (7)	0.0324 (16)	0.1929 (17)
F3X	−0.1326 (12)	0.6656 (11)	0.1492 (5)	0.077 (3)	0.1929 (17)
F4X	0.1509 (10)	0.6010 (6)	0.2425 (4)	0.0288 (12)	0.1929 (17)
F5X	0.2115 (10)	0.6342 (6)	0.0790 (5)	0.0465 (4)	0.1929 (17)
F6X	−0.0153 (13)	0.8381 (7)	0.2262 (5)	0.0484 (4)	0.1929 (17)
N1	0.46336 (19)	0.22118 (13)	0.12431 (7)	0.0216 (2)	
N2	0.6288 (2)	0.30531 (13)	0.23067 (8)	0.0233 (2)	
N3	0.8071 (2)	−0.08439 (15)	0.65425 (9)	0.0290 (2)	
C1	0.4607 (2)	0.33312 (14)	0.17476 (9)	0.0229 (2)	
H1A	0.3558	0.4190	0.1715	0.027*	
C2	0.6385 (2)	0.11742 (16)	0.14906 (9)	0.0267 (3)	
H2A	0.6791	0.0262	0.1240	0.032*	
C3	0.7419 (2)	0.16950 (17)	0.21571 (9)	0.0270 (3)	
H3A	0.8686	0.1217	0.2465	0.032*	
C4	0.6750 (3)	0.39959 (17)	0.30071 (10)	0.0312 (3)	
H4A	0.6125	0.5067	0.2803	0.037*	
H4B	0.8399	0.4054	0.3004	0.037*	
C5	0.5724 (2)	0.33138 (14)	0.40301 (9)	0.0238 (2)	
C6	0.3613 (3)	0.38393 (16)	0.43888 (10)	0.0278 (3)	
H6A	0.2810	0.4643	0.3991	0.033*	
C7	0.2671 (2)	0.31960 (17)	0.53257 (11)	0.0288 (3)	
H7A	0.1238	0.3572	0.5565	0.035*	
C8	0.3807 (2)	0.20105 (16)	0.59146 (10)	0.0253 (3)	
H8A	0.3163	0.1570	0.6554	0.030*	
C9	0.5913 (2)	0.14774 (15)	0.55501 (9)	0.02253 (17)	
C10	0.6883 (2)	0.21320 (15)	0.46148 (9)	0.0222 (2)	
H10A	0.8329	0.1771	0.4379	0.027*	
C11	0.3054 (3)	0.21229 (18)	0.05341 (10)	0.0303 (3)	
H11A	0.1951	0.2996	0.0509	0.045*	
H11B	0.2269	0.1141	0.0732	0.045*	
H11C	0.3889	0.2172	−0.0112	0.045*	
C12	0.7105 (2)	0.01965 (15)	0.61205 (9)	0.02253 (17)	

### Atomic displacement parameters (Å^2^)

**Table:**

	*U*^11^	*U*^22^	*U*^33^	*U*^12^	*U*^13^	*U*^23^
P1	0.0196 (5)	0.0133 (3)	0.0188 (3)	0.0025 (3)	−0.0025 (3)	−0.0035 (3)
F1	0.0187 (5)	0.0593 (9)	0.0658 (10)	−0.0012 (6)	0.0014 (5)	−0.0109 (8)
F2	0.0589 (15)	0.0616 (16)	0.0208 (7)	−0.0189 (10)	−0.0030 (8)	0.0067 (8)
F3	0.0226 (5)	0.0446 (7)	0.0422 (7)	0.0100 (5)	−0.0036 (5)	−0.0058 (6)
F4	0.0442 (9)	0.0523 (10)	0.0318 (8)	−0.0136 (7)	−0.0165 (6)	0.0179 (7)
F5	0.0475 (8)	0.0333 (6)	0.0690 (9)	0.0102 (5)	−0.0183 (7)	−0.0302 (6)
F6	0.0702 (10)	0.0290 (6)	0.0559 (8)	0.0030 (6)	−0.0275 (8)	−0.0211 (6)
P1X	0.019 (3)	0.053 (4)	0.058 (4)	0.001 (2)	−0.007 (2)	0.000 (3)
F1X	0.035 (3)	0.060 (4)	0.047 (3)	−0.026 (3)	−0.014 (2)	0.010 (3)
F2X	0.036 (4)	0.023 (3)	0.028 (3)	0.010 (2)	0.006 (2)	0.008 (2)
F3X	0.047 (4)	0.112 (7)	0.055 (4)	−0.054 (5)	−0.038 (3)	0.056 (5)
F4X	0.039 (3)	0.027 (2)	0.015 (2)	0.012 (2)	−0.0012 (18)	0.0023 (17)
F5X	0.0475 (8)	0.0333 (6)	0.0690 (9)	0.0102 (5)	−0.0183 (7)	−0.0302 (6)
F6X	0.0702 (10)	0.0290 (6)	0.0559 (8)	0.0030 (6)	−0.0275 (8)	−0.0211 (6)
N1	0.0237 (5)	0.0234 (5)	0.0160 (4)	0.0022 (4)	−0.0016 (4)	−0.0014 (4)
N2	0.0265 (5)	0.0237 (5)	0.0181 (4)	−0.0015 (4)	−0.0018 (4)	−0.0006 (4)
N3	0.0273 (6)	0.0320 (6)	0.0274 (5)	0.0024 (5)	−0.0061 (4)	−0.0048 (5)
C1	0.0261 (6)	0.0206 (5)	0.0200 (5)	0.0030 (4)	−0.0013 (4)	−0.0012 (4)
C2	0.0310 (6)	0.0268 (6)	0.0196 (5)	0.0097 (5)	0.0008 (5)	−0.0035 (4)
C3	0.0251 (6)	0.0329 (6)	0.0198 (5)	0.0077 (5)	−0.0013 (4)	−0.0011 (5)
C4	0.0437 (8)	0.0280 (6)	0.0221 (6)	−0.0134 (6)	−0.0048 (5)	−0.0014 (5)
C5	0.0313 (6)	0.0211 (5)	0.0203 (5)	−0.0053 (5)	−0.0054 (5)	−0.0045 (4)
C6	0.0348 (7)	0.0232 (5)	0.0273 (6)	0.0041 (5)	−0.0112 (5)	−0.0068 (5)
C7	0.0285 (6)	0.0296 (6)	0.0297 (6)	0.0070 (5)	−0.0034 (5)	−0.0111 (5)
C8	0.0268 (6)	0.0275 (6)	0.0217 (5)	0.0018 (5)	−0.0006 (5)	−0.0074 (5)
C9	0.0234 (4)	0.0245 (4)	0.0208 (4)	−0.0005 (3)	−0.0034 (3)	−0.0066 (3)
C10	0.0222 (5)	0.0242 (5)	0.0213 (5)	−0.0022 (4)	−0.0029 (4)	−0.0067 (4)
C11	0.0322 (7)	0.0357 (7)	0.0237 (6)	−0.0039 (6)	−0.0067 (5)	−0.0046 (5)
C12	0.0234 (4)	0.0245 (4)	0.0208 (4)	−0.0005 (3)	−0.0034 (3)	−0.0066 (3)

### Geometric parameters (Å, °)

**Table:**

P1—F2	1.571 (2)	C2—C3	1.351 (2)
P1—F1	1.5860 (17)	C2—H2A	0.9500
P1—F3	1.5964 (17)	C3—H3A	0.9500
P1—F6	1.5983 (15)	C4—C5	1.5141 (19)
P1—F5	1.5993 (14)	C4—H4A	0.9900
P1—F4	1.6257 (18)	C4—H4B	0.9900
P1X—F6X	1.523 (8)	C5—C10	1.3899 (18)
P1X—F4X	1.552 (8)	C5—C6	1.394 (2)
P1X—F3X	1.560 (8)	C6—C7	1.391 (2)
P1X—F1X	1.598 (8)	C6—H6A	0.9500
P1X—F2X	1.619 (10)	C7—C8	1.3875 (19)
P1X—F5X	1.637 (8)	C7—H7A	0.9500
N1—C1	1.3275 (17)	C8—C9	1.3959 (18)
N1—C2	1.3751 (17)	C8—H8A	0.9500
N1—C11	1.4695 (18)	C9—C10	1.3970 (18)
N2—C1	1.3298 (17)	C9—C12	1.4444 (18)
N2—C3	1.3799 (17)	C10—H10A	0.9500
N2—C4	1.4733 (18)	C11—H11A	0.9800
N3—C12	1.1471 (18)	C11—H11B	0.9800
C1—H1A	0.9500	C11—H11C	0.9800
			
F2—P1—F1	92.46 (13)	N2—C1—H1A	125.6
F2—P1—F3	90.74 (12)	C3—C2—N1	107.16 (12)
F1—P1—F3	176.79 (11)	C3—C2—H2A	126.4
F2—P1—F6	91.41 (12)	N1—C2—H2A	126.4
F1—P1—F6	90.49 (11)	C2—C3—N2	107.07 (12)
F3—P1—F6	89.64 (10)	C2—C3—H3A	126.5
F2—P1—F5	91.54 (13)	N2—C3—H3A	126.5
F1—P1—F5	91.14 (10)	N2—C4—C5	111.59 (11)
F3—P1—F5	88.56 (10)	N2—C4—H4A	109.3
F6—P1—F5	176.57 (12)	C5—C4—H4A	109.3
F2—P1—F4	179.81 (15)	N2—C4—H4B	109.3
F1—P1—F4	87.62 (10)	C5—C4—H4B	109.3
F3—P1—F4	89.18 (10)	H4A—C4—H4B	108.0
F6—P1—F4	88.76 (10)	C10—C5—C6	119.47 (12)
F5—P1—F4	88.29 (10)	C10—C5—C4	119.72 (13)
F6X—P1X—F4X	93.3 (5)	C6—C5—C4	120.81 (13)
F6X—P1X—F3X	92.6 (6)	C7—C6—C5	120.48 (12)
F4X—P1X—F3X	92.2 (5)	C7—C6—H6A	119.8
F6X—P1X—F1X	91.3 (5)	C5—C6—H6A	119.8
F4X—P1X—F1X	91.2 (5)	C8—C7—C6	120.60 (13)
F3X—P1X—F1X	174.7 (6)	C8—C7—H7A	119.7
F6X—P1X—F2X	90.2 (5)	C6—C7—H7A	119.7
F4X—P1X—F2X	176.5 (6)	C7—C8—C9	118.78 (12)
F3X—P1X—F2X	87.9 (6)	C7—C8—H8A	120.6
F1X—P1X—F2X	88.5 (6)	C9—C8—H8A	120.6
F6X—P1X—F5X	176.9 (6)	C8—C9—C10	120.97 (12)
F4X—P1X—F5X	89.9 (4)	C8—C9—C12	120.61 (12)
F3X—P1X—F5X	87.1 (6)	C10—C9—C12	118.39 (12)
F1X—P1X—F5X	88.8 (5)	C5—C10—C9	119.70 (12)
F2X—P1X—F5X	86.6 (5)	C5—C10—H10A	120.2
C1—N1—C2	108.59 (11)	C9—C10—H10A	120.2
C1—N1—C11	125.08 (11)	N1—C11—H11A	109.5
C2—N1—C11	126.33 (12)	N1—C11—H11B	109.5
C1—N2—C3	108.33 (11)	H11A—C11—H11B	109.5
C1—N2—C4	125.08 (12)	N1—C11—H11C	109.5
C3—N2—C4	126.49 (12)	H11A—C11—H11C	109.5
N1—C1—N2	108.85 (11)	H11B—C11—H11C	109.5
N1—C1—H1A	125.6	N3—C12—C9	177.42 (14)
			
C2—N1—C1—N2	0.34 (14)	N2—C4—C5—C6	−95.13 (16)
C11—N1—C1—N2	−179.13 (12)	C10—C5—C6—C7	0.3 (2)
C3—N2—C1—N1	−0.48 (14)	C4—C5—C6—C7	179.33 (13)
C4—N2—C1—N1	−177.01 (11)	C5—C6—C7—C8	−0.7 (2)
C1—N1—C2—C3	−0.07 (15)	C6—C7—C8—C9	0.1 (2)
C11—N1—C2—C3	179.40 (12)	C7—C8—C9—C10	0.8 (2)
N1—C2—C3—N2	−0.22 (15)	C7—C8—C9—C12	−177.06 (13)
C1—N2—C3—C2	0.43 (15)	C6—C5—C10—C9	0.52 (19)
C4—N2—C3—C2	176.90 (12)	C4—C5—C10—C9	−178.47 (12)
C1—N2—C4—C5	95.87 (16)	C8—C9—C10—C5	−1.09 (19)
C3—N2—C4—C5	−80.04 (18)	C12—C9—C10—C5	176.79 (12)
N2—C4—C5—C10	83.85 (16)		

### Hydrogen-bond geometry (Å, °)

**Table:**

*D*—H···*A*	*D*—H	H···*A*	*D*···*A*	*D*—H···*A*
C1—H1A···F1	0.95	2.46	3.240 (2)	139
C1—H1A···F5	0.95	2.27	3.1715 (18)	159
C2—H2A···F3^i^	0.95	2.46	3.250 (2)	140
C3—H3A···N3^ii^	0.95	2.49	3.3970 (19)	160
C4—H4B···F4^iii^	0.99	2.44	3.177 (2)	131
C6—H6A···F4	0.95	2.43	3.361 (2)	167
C10—H10A···N3^ii^	0.95	2.56	3.5019 (17)	170
C11—H11B···F6^iv^	0.98	2.53	3.400 (2)	148
C11—H11C···F1^v^	0.98	2.41	3.353 (2)	162

Symmetry codes: (i) *x*+1, *y*−1, *z*; (ii) −*x*+2, −*y*, −*z*+1; (iii) *x*+1, *y*, *z*; (iv) *x*, *y*−1, *z*; (v) −*x*+1, −*y*+1, −*z*.
